# Dynamic hypernode injection and graph attention fusion for multimodal sentiment analysis of behavioral signals

**DOI:** 10.1371/journal.pone.0359425

**Published:** 2026-09-28

**Authors:** Wenfei Cao, Jian Xu, Yinghao Li, Huizi Yan, Zhuowei Hu, Wenxu Chen

**Affiliations:** College of Intelligent Systems Science and Engineering, Hubei Minzu University, Enshi, China; Hunan Normal University, CHINA

## Abstract

Language use, vocal behavior, and facial activity provide complementary indicators of affective state, but multimodal models can be affected by noisy temporal observations and insufficient global guidance before cross-modal interaction. We developed a framework combining bidirectional gated recurrent unit encoders, attention pooling, dynamic hypernode injection, and graph attention fusion. Textual, acoustic, and visual sequences were mapped into a shared latent space and compressed into modality-level representations. A sample-dependent hypernode and a learnable static prior were then injected through gated residual connections before graph propagation. The model was evaluated on CMU-MOSI and CMU-MOSEI using five random seeds and validation-MAE checkpoint selection. On CMU-MOSI, the model obtained an MAE of 0.7290±0.0099, a correlation of 0.7880±0.0076, and an Acc-7 of 0.4561±0.0160. On CMU-MOSEI, the corresponding results were 0.5541±0.0053, 0.7551±0.0058, and 0.5269±0.0064. A five-run comparison of pre-GAT, post-GAT, and no injection showed that pre-GAT injection provided the strongest overall regression-oriented balance, although post-GAT was slightly higher on MOSEI Acc-7. Gate, graph-attention, representation, and balanced case diagnostics showed that the injected prior was sample dependent and modality selective. These findings support pre-propagation hypernode injection under complete, word-aligned tri-modal inputs, without establishing universal superiority or robustness to missing and corrupted modalities.

## Introduction

Observable language use, vocal patterns, and facial behavior provide complementary information about affective state and interpersonal expression. Computational models that integrate these signals are increasingly used in affective computing and behavior-aware human-computer interaction. The affective computing framework [1] laid the theoretical foundation for this field. Subsequent work showed that jointly modeling facial expressions, body movements, and speech information can improve emotion recognition [2]. More recently, a comprehensive review [3] surveyed multimodal emotion recognition across theoretical foundations, datasets, modality-specific feature extraction, cross-modal alignment, and fusion strategies, and summarized the strengths, limitations, challenges, and future directions of the field.

CMU-MOSI [4] and CMU-MOSEI [5] are standard video-based benchmarks for multimodal sentiment analysis. Here they are used to examine a modeling question rather than as the motivation by themselves: how sample-level global information can guide structured cross-modal propagation while modality-specific temporal evidence is retained.

As multimodal fusion methods have continued to evolve, tensor fusion has been used to explicitly model higher-order interactions among text, acoustic, and visual modalities [6], multi-view memory mechanisms have been applied to capture cross-modal dynamics [7], and low-rank decomposition has been employed to reduce the computational cost of fusion [8]. Transformer-based and representation-learning approaches have further improved cross-modal interaction, including directional cross-modal attention for unaligned sequences [9] and modality-invariant and modality-specific representation learning [10]. Cross-modal enhancement networks have been designed to enrich textual representations with non-textual information [11], and text-enhanced transformer fusion has been developed to emphasize the guiding role of language in sentiment prediction [12]. Recent studies have also examined sequential fusion of text-close and text-far representations [13], multimodal adapter mixtures [14], invariant multimodal representations [15], and directed pairwise cross-modal GRU attention [16]. Complementarity- and importance-aware fusion has also been proposed for multimodal emotion recognition [17], while fuzzy cognition-based dynamic fusion has been used to integrate modality-specific sentiment scores for multimodal sentiment analysis [18].

Beyond conventional fusion, structured modeling has become an important direction for capturing complex multimodal dependencies. DialogueGCN [19] and COGMEN [20] obtain contextual information from utterance-level conversational graphs, speaker relations, and message passing. ALMT [21] instead constructs a language-guided adaptive hyper-modality during staged cross-modal interaction. These mechanisms are effective for their respective settings, but they do not explicitly construct a sample-specific tri-modal prior and inject it into every modality node before the first graph-attention layer. The proposed pipeline makes this ordering explicit while retaining a compact three-node segment graph. Table 1 summarizes these structural differences.

**Table 1:** Structural comparison with representative global and graph-based fusion mechanisms.

Method	Global/context mechanism	Where context enters	Primary graph or fusion unit
ALMT [21]	Language-guided adaptive hyper-modality	During staged cross-modal interaction	Multimodal feature sequences
DialogueGCN [19]	Speaker and conversational context	Through graph message passing	Utterance nodes in dialogue
COGMEN [20]	Contextualized multimodal graph	Through multimodal conversational propagation	Utterance and relation structure
Proposed method	Dynamic tri-modal hypernode plus static prior	Gated residual injection before the first GAT layer	Three modality nodes for one segment

Despite these advances, three modeling gaps remain relevant. First, heterogeneous modality features may enter fusion without a sufficiently comparable latent representation. Second, sample-level global information is not always used to condition all modality nodes before structured propagation begins. Third, redundant temporal observations can enter graph reasoning unless sequence evidence is compressed before cross-modal interaction.

To address these issues, we propose a multimodal sentiment analysis framework that integrates BiGRU-based intra-modal temporal modeling, dynamic hypernode injection, and graph attention fusion. Specifically, the proposed method first maps textual, acoustic, and visual features into a shared latent space through a text encoder and modality-specific linear projections. It then employs BiGRU and attention pooling to obtain modality-level node representations that preserve temporal dynamics while suppressing irrelevant time-step noise. On this basis, a dynamic hypernode is constructed from the global tri-modal context and combined with a learnable static hyper-prior. The resulting global semantic prior is adaptively injected into each modality node through a gated residual mechanism before cross-modal propagation. Finally, the enhanced modality nodes are organized as a fully connected tri-modal graph, and graph attention fusion is used to model structured inter-modal dependencies for continuous sentiment intensity prediction.

By explicitly introducing global semantic guidance before graph propagation and combining it with modality-aware temporal compression, the proposed framework provides a structurally distinct fusion pipeline. Experiments on CMU-MOSI and CMU-MOSEI evaluate this design across regression and classification metrics, five random seeds, three injection positions, component ablations, and direct mechanism diagnostics. The contribution is architectural and empirical within complete, aligned tri-modal inputs; it is not a claim of universal robustness.

## Materials and methods

### Study design and data sources

This computational benchmark study evaluates a multimodal sentiment model using two public datasets, CMU-MOSI and CMU-MOSEI. CMU-MOSI [4] contains 2,199 opinion-level video segments, and CMU-MOSEI [5] contains 22,856 segments with a broader speaker distribution and more diverse scenarios. Both benchmarks use continuous sentiment scores in the range [−3,3]; these scores also support the seven ordered classes used for Acc-7. Table 2 summarizes the official training, validation, and test partitions used in this study.

**Table 2:** Dataset partitions and label information used in the experiments.

Dataset	Total clips	Train	Validation	Test	Label range	Source / split provenance
CMU-MOSI	2,199	1,284	229	686	[−3,3]	CMU-MOSI [4]; official train/validation/test split
CMU-MOSEI	22,856	16,326	1,871	4,659	[−3,3]	CMU-MOSEI [5]; official train/validation/test split

Both datasets provide textual, acoustic, and visual modalities and are used here as established benchmarks of affect-related behavioral signal modeling.

### Overall framework

For continuous sentiment-intensity prediction, let the text, acoustic, and visual inputs be $X^{t}\in {\mathbb{R}}^{T_{t}\times d_{t}}$, $X^{a}\in {\mathbb{R}}^{T_{a}\times d_{a}}$, and $X^{v}\in {\mathbb{R}}^{T_{v}\times d_{v}}$, respectively. Here, $T_{m}$ is the sequence length of modality *m*, $d_{m}$ is its input dimension, and y∈ℝ is the sentiment label. The objective is to learn the following end-to-end mapping:


$$\begin{array}{c} \hat{y}=f(X^{t},X^{a},X^{v}) \end{array}$$ (1)

where $\hat{y}$ denotes the predicted sentiment intensity.

To jointly model the complementary information conveyed by temporal dynamics, global context, and cross-modal dependencies, we propose a multimodal sentiment analysis framework based on dynamic hypernode injection and graph attention fusion. The overall pipeline consists of five stages. First, a pretrained language model and modality-specific linear projections are used to map the three modalities into a shared latent space. Second, bidirectional gated recurrent units [22] are employed to model intra-modal temporal dependencies, and attention pooling is used to obtain modality-level node representations. Third, a dynamic hypernode is constructed from the global tri-modal context and injected into each modality node through a gated residual mechanism. Fourth, multi-layer graph attention networks [23] are applied to a cross-modal graph composed of text, acoustic, and visual nodes in order to perform structured relation propagation. Finally, the graph-fused representation is fed into a regression head to predict sentiment intensity.

As illustrated in Fig 1, the proposed framework follows a hierarchical modeling process from single-modal representation learning to global interaction and downstream prediction. The three modalities are first aligned in a unified latent space and then compressed into modality-level nodes through temporal encoding and attention pooling. These nodes are subsequently enhanced by dynamic hypernode injection. On this basis, cross-modal dependencies are modeled through graph attention propagation over a fully connected three-node graph, and the final sentiment intensity score is obtained through node-level readout and regression. Table 3 summarizes the principal symbols used in the model formulation.

**Fig 1:**
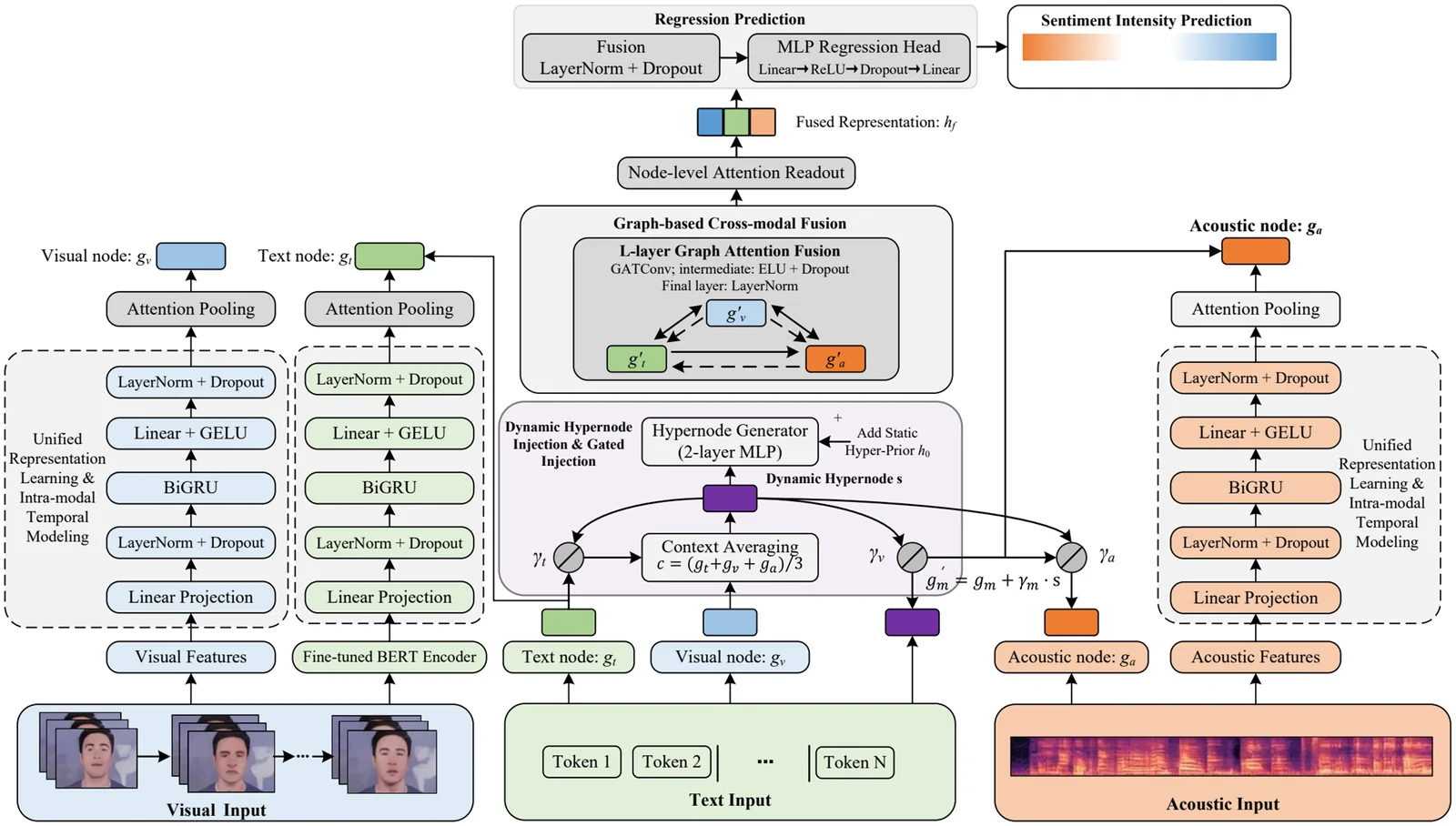
Multimodal sentiment analysis framework based on dynamic hypernode injection and graph attention fusion. The figure was created by the authors. The face sequence in the visual-input block is a schematic illustration and does not depict a study participant or any identifiable individual.

**Table 3:** Principal notation used in the model formulation.

Symbol	Definition	Symbol	Definition
m∈{t,a,v}	Text, acoustic, or visual modality	$T_{m},d_{m}$	Sequence length and input dimension
*d*	Shared latent dimension	$g^{m}$	Pooled modality node
*c*	Mean tri-modal context	*s*	Dynamic hypernode plus static prior
$\gamma^{m}$	Modality-specific injection gate	$h_{i}^{\left(l\right)}$	Node *i* at graph layer *l*
$\alpha_{ij}^{\left(l\right)}$	Graph-attention coefficient	$\beta_{i}$	Node-readout weight
$h_{f}$	Graph-level fused representation	$\hat{y},y$	Predicted and reference sentiment scores

### Unified modal representation learning

For the text modality, we use a pretrained BERT encoder [24] to extract contextualized semantic representations. Specifically, the textual input consists of input_ids, attention_mask, and token_type_ids, and the final hidden states produced by BERT are taken as the text sequence representation. In implementation, the BERT parameters are fine-tuned end to end to improve task-specific adaptability.

Let the text representation after BERT encoding be $H^{t}$. For *m* = *t*, $X^{t}=H^{t}\in {\mathbb{R}}^{T_{t}\times d_{t}}$; for *m* = *a*, $X^{a}\in {\mathbb{R}}^{T_{a}\times d_{a}}$; and for *m* = *v*, $X^{v}\in {\mathbb{R}}^{T_{v}\times d_{v}}$. To reduce modality heterogeneity, we assign each modali*t*y an independent projection and map all modalities into a shared latent space of dimension *d*:


$$\begin{array}{c} Z^{m}=W_{m}X^{m}+b_{m},\;m\in \{t,a,v\} \end{array}$$ (2)

where *m* = *t*, $X^{t}=H^{t}$ for the text modality. After projection, layer normalization and dropout are further applied to improve training stability and generalization:


$$\begin{array}{c} {\tilde{Z}}^{m}=\text{Dropout}(\text{LN}(Z^{m})) \end{array}$$ (3)

Through this process, the text, acoustic, and visual modalities are brought into the same representational space, providing comparable inputs for subsequent intra-modal temporal modeling and cross-modal graph fusion.

### Intra-modal sequence modeling

To capture dynamic dependencies within each modality, we encode the unified text, acoustic, and visual sequences with bidirectional gated recurrent units. For modality *m*, given ${\tilde{z}}_{i}^{m}$ at time step *i*, the forward and backward hidden states are computed as:


$$\begin{array}{c} {\vec{h}}_{i}^{m}={\text{GRU}}_{f}({\tilde{z}}_{i}^{m},{\vec{h}}_{i-1}^{m}) \end{array}$$ (4)

$$\begin{array}{c} {\overset{\leftarrow }{h}}_{i}^{m}={\text{GRU}}_{b}({\tilde{z}}_{i}^{m},{\overset{\leftarrow }{h}}_{i+1}^{m}) \end{array}$$ (5)

By concatenating the bidirectional outputs, we obtain the temporal feature representation:


$$\begin{array}{c} h_{i}^{m}=[{\vec{h}}_{i}^{m};{\overset{\leftarrow }{h}}_{i}^{m}] \end{array}$$ (6)

Since the bidirectional encoding expands the hidden dimension to 2*d*, we compress it back to *d* dimensions through a linear projection, followed by GELU, layer normalization, and dropout:


$$\begin{array}{c} s_{i}^{m}=\text{Dropout}\left(\text{LN}(\text{GELU}(W_{p}^{m}h_{i}^{m}+b_{p}^{m}))\right) \end{array}$$ (7)

To compress each aligned sequence into a modality-level node representation, we use linear attention pooling. For the *i*-th time step of modality *m*, the attention score is defined as:


$$\begin{array}{c} u_{i}^{m}={w_{\text{att}}^{m}}^{⊤}s_{i}^{m}+b_{\text{att}}^{m} \end{array}$$ (8)

The normalized weights are:


$$\begin{array}{c} \alpha_{i}^{m}=\frac{\exp(u_{i}^{m})}{\sum_{k=1}^{T_{m}}\exp(u_{k}^{m})} \end{array}$$ (9)

The final modality-level node representation is given by:


$$\begin{array}{c} g^{m}=\sum_{i=1}^{T_{m}}\alpha_{i}^{m}s_{i}^{m} \end{array}$$ (10)

This BiGRU-plus-attention-pooling design combines sequential encoding with learned temporal weighting. Its contribution is evaluated by the repeated-seed ablation results below.

### Dynamic hypernode construction and gated injection

Relying solely on pairwise interactions between modalities often makes it difficult to introduce a global semantic prior before cross-modal propagation. To address this issue, we design a dynamic hypernode module that aggregates the global context of the three modality nodes for the current sample and injects it into each modality representation as prior information.

First, the text, acoustic, and visual modality nodes are averaged to obtain a sample-level context vector:


$$\begin{array}{c} c=\frac{g^{t}+g^{a}+g^{v}}{3} \end{array}$$ (11)

Next, a two-layer multilayer perceptron performs a nonlinear transformation of the global context and generates the hypernode representation:


$$\begin{array}{c} \tilde{s}=\text{MLP}(c) \end{array}$$ (12)

Unlike a purely input-driven dynamic representation, we further introduce a learnable global prior vector $h_{0}\in {\mathbb{R}}^{d}$ as a bias term. In the implementation, this prior is derived from the first token of the learnable hypernode parameters. The final dynamic hypernode representation is therefore written as:


$$\begin{array}{c} s=\tilde{s}+h_{0} \end{array}$$ (13)

To avoid imposing an identical degree of global injection on all modalities, we assign each modality an individual scalar gating coefficient to control the influence of the hypernode on that modality. For modality *m*, the gating value is defined as


$$\begin{array}{c} \gamma^{m}=\sigma ({w_{g}^{m}}^{⊤}g^{m}+b_{g}^{m}) \end{array}$$ (14)

where $w_{g}^{m}\in {\mathbb{R}}^{d}$ and $b_{g}^{m}\in \mathbb{R}$ are learnable gate parameters and σ(·) denotes the sigmoid function. Finally, the global prior is injected through a residual formulation:


$$\begin{array}{c} {\overset{―}{g}}^{m}=g^{m}+\gamma^{m}s,\;m\in \{t,a,v\} \end{array}$$ (15)

Through this design, the model can adaptively inject the global semantic prior jointly formed by the three modalities into each modality representation while preserving modality-specific characteristics, thereby alleviating multimodal information fragmentation and insufficient global context modeling.

### Graph-based attention for cross-modal fusion

After dynamic hypernode injection, the hypernode is not directly introduced as an additional graph node. Instead, as illustrated in Fig 2, it is generated once from the tri-modal context together with the static hyper-prior and is used to enhance the three modality nodes before graph propagation. Let $g_{t}^{\prime },g_{a}^{\prime },g_{v}^{\prime }\in {\mathbb{R}}^{d}$ denote the enhanced text, acoustic, and visual nodes, respectively. We then construct a fully connected tri-modal graph *G* = (*V*, *E*), where $𝒱=\{g_{t}^{\prime },g_{a}^{\prime },g_{v}^{\prime }\}$. This design keeps the graph structure compact while allowing the injected global prior to participate in subsequent cross-modal propagation through the enhanced modality nodes. As a result, the graph explicitly represents the latent dependencies among the three modalities and provides a structured basis for cross-modal information fusion.

**Fig 2:**
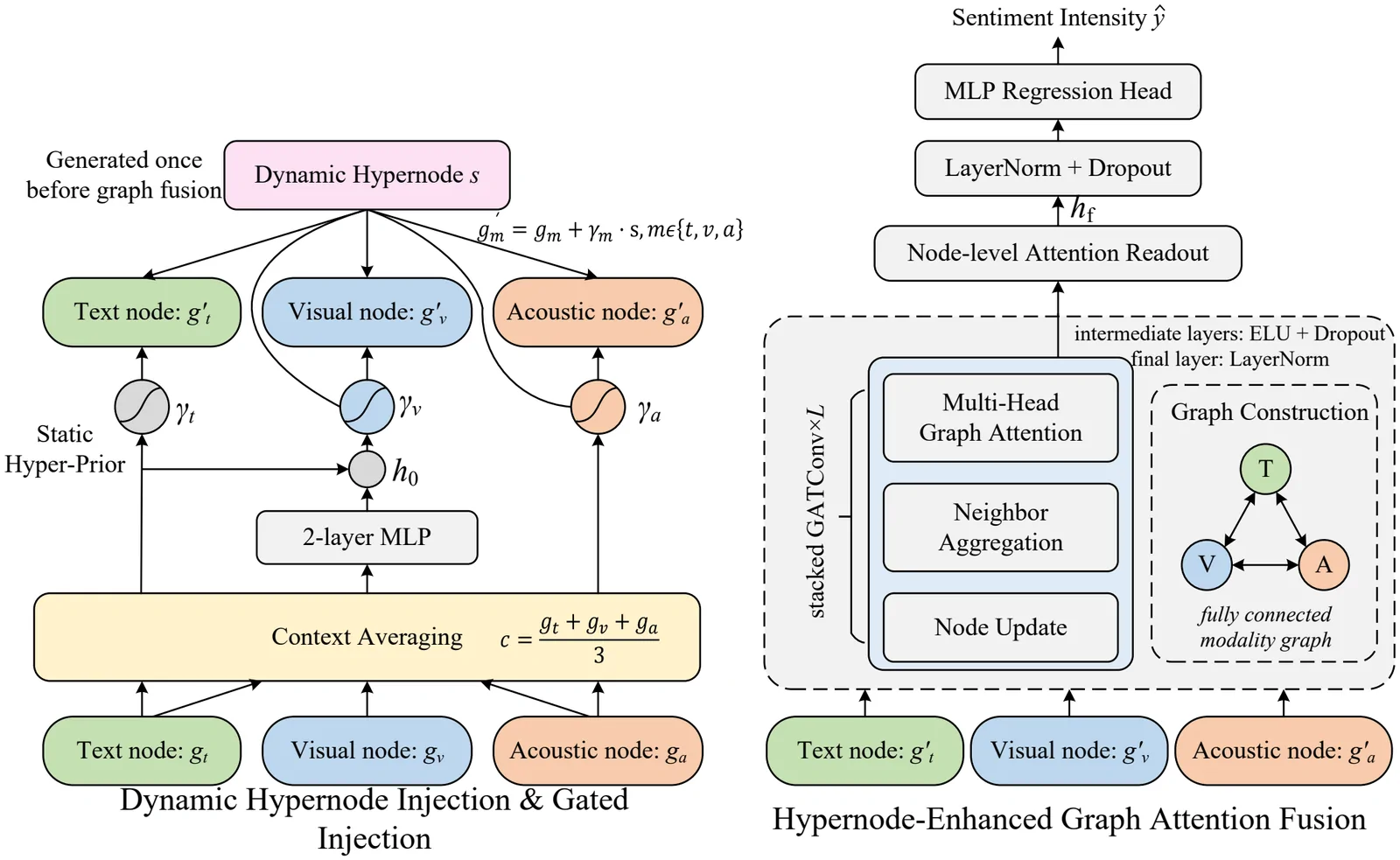
Dynamic hypernode injection and graph-attention fusion. The sample-level context is transformed into a hypernode, combined with a static prior, and added to each modality through a learned gate. The enhanced text, acoustic, and visual nodes form a fully connected three-node graph; the hypernode itself is not an additional graph node.

For convenience, the initial node states are defined as:


$$\begin{array}{c} h_{t}^{\left(0\right)}=g_{t}^{\prime },\;h_{a}^{\left(0\right)}=g_{a}^{\prime },\;h_{v}^{\left(0\right)}=g_{v}^{\prime } \end{array}$$ (16)

During graph propagation, we employ stacked graph attention layers to model inter-modal interactions. In the *l*-th layer, the unnormalized attention coefficient between node *i* and its neighbor j∈𝒩(i) is computed as


$$\begin{array}{c} e_{ij}^{\left(l\right)}=\text{LeakyReLU}\left({a^{\left(l\right)}}^{⊤}\left[W^{\left(l\right)}h_{i}^{\left(l\right)}\|W^{\left(l\right)}h_{j}^{\left(l\right)}\right]\right) \end{array}$$ (17)

where *W*^(*l*)^ is a learnable linear transformation matrix, *a*^(*l*)^ is the attention vector, and ‖ denotes vector concatenation. The corresponding normalized attention weight is:


$$\begin{array}{c} \alpha_{ij}^{\left(l\right)}=\frac{\exp(e_{ij}^{\left(l\right)})}{\sum_{k\in 𝒩(i)}\exp(e_{ik}^{\left(l\right)})} \end{array}$$ (18)

The node update process can be written as:


$$\begin{array}{c} h_{i}^{\left(l+1\right)}=\sigma \left(\sum_{j\in 𝒩(i)}\alpha_{ij}^{\left(l\right)}W^{\left(l\right)}h_{j}^{\left(l\right)}\right) \end{array}$$ (19)

where σ(·) denotes the non-linear activation function. Through this layer-wise aggregation mechanism, each modality node can adaptively absorb information from the other modalities according to sample-specific relational strength, thereby enabling structured cross-modal interaction rather than simple feature concatenation.

To model deeper inter-modal dependency propagation, the graph fusion block stacks $L_{g}$ graph attention layers. Each non-final layer is followed by ELU and Dropout to enhance non-linear expressiveness and reduce overfitting, whereas LayerNorm is applied after the final layer to stabilize node representations after deep graph propagation. After the final layer, we obtain the node set $\left\{h_{t}^{\left(L_{g}\right)},h_{a}^{\left(L_{g}\right)},h_{v}^{\left(L_{g}\right)}\right\}$. We then perform node-level attention pooling for graph readout. Specifically, an importance score is assigned to each modality node as:


$$\begin{array}{c} r_{i}=w_{r}^{⊤}h_{i}^{\left(L_{g}\right)}+b_{r},\;i\in \{t,a,v\} \end{array}$$ (20)

and the corresponding readout weight is computed by


$$\begin{array}{c} \beta_{i}=\frac{\exp(r_{i})}{\sum_{j\in \{t,a,v\}}\exp(r_{j})} \end{array}$$ (21)

The final fused representation is given by


$$\begin{array}{c} h_{f}=\sum_{i\in \{t,a,v\}}\beta_{i}h_{i}^{\left(L_{g}\right)} \end{array}$$ (22)

This graph attention fusion mechanism enables the model to propagate and aggregate cross-modal information according to the contribution of each modality in a given sample, thereby producing a fused representation that is both globally coherent and cross-modally complementary.

### Sentiment regression prediction and model training

Following graph readout, the fused graph-level representation $h_{f}$ is first regularized by layer normalization and dropout:


$$\begin{array}{c} z_{f}=\text{Dropout}(\text{LN}(h_{f})) \end{array}$$ (23)

A two-layer multi-layer perceptron is then used as the regression head to predict continuous sentiment intensity. The prediction process is formulated as:


$$\begin{array}{c} o=\text{ReLU}(W_{1}z_{f}+b_{1}) \end{array}$$ (24)

$$\begin{array}{c} \hat{y}=W_{2}(\text{Dropout}(o))+b_{2} \end{array}$$ (25)

where $W_{1},b_{1},W_{2},b_{2}$ are the two regression-layer parameters, *o* is the hidden prediction vector, and $\hat{y}$ is the scalar sentiment score. This design is consistent with the regression branch shown on the right-hand side of Fig 2, where the graph-level fused representation is transformed into a scalar sentiment-intensity prediction.

For the continuous sentiment intensity prediction tasks on CMU-MOSI and CMU-MOSEI, the model is optimized using mean squared error:


$$\begin{array}{c} {ℒ}_{\text{MSE}}=\frac{1}{N}\sum_{n=1}^{N}({\hat{y}}_{n}-y_{n}{)}^{2} \end{array}$$ (26)

where *N* is the number of samples in a mini-batch and $y_{n}$ is the ground-truth sentiment label of the *n*-th sample.

During training, the entire framework is optimized end to end with AdamW. Unified modal representation learning, intra-modal temporal modeling, dynamic hypernode injection, graph-based cross-modal fusion, and sentiment regression are jointly learned within a single objective.

### Implementation details

The model is implemented in PyTorch [25]. The archived environment uses Python 3.10.0, PyTorch 2.4.1 + cu118, CUDA 11.8, cuDNN 90100, and one NVIDIA RTX 4080 SUPER GPU. The ALMT training program loads word-aligned serialized feature artifacts named aligned_50.pkl. Text is encoded with bert-base-uncased and has an output dimension of 768; the aligned acoustic and visual features have 25 and 171 dimensions, respectively. Fixed sequence lengths are 45 for MOSI and 75 for MOSEI. The exact CMU-MultimodalSDK release/commit and original acoustic/visual extractor versions are not preserved in the supplied records and are therefore reported as unavailable rather than inferred. The training and analysis code used for this study is publicly archived at Zenodo (https://doi.org/10.5281/zenodo.22218345) [26].

At runtime, the text tensor has three channels for input IDs, attention mask, and token-type IDs. Acoustic negative-infinity values are replaced with zero, no additional feature normalization is applied, and all tensors are converted to float32.

The model is trained with mean squared error and AdamW [27]. The fixed settings are batch size 64, learning rate 10^–4^, weight decay 10^–4^, and a maximum of 200 epochs. Early stopping uses a patience of 40 epochs, and the checkpoint with the lowest validation MAE is selected. Seven graph-attention layers are used for MOSI and four for MOSEI. Five random seeds are run for the main model, comparison methods, component ablations, and each injection-position condition. For these experiments, the reported rows give means and sample standard deviations across five runs, and every metric within a run comes from the same validation-selected checkpoint. Table 4 consolidates the principal feature, training, and runtime settings. Except for the fixed sequence length and the graph depth selected from the development-stage depth sweep, the listed settings were kept the same for MOSI and MOSEI.

**Table 4:** Principal implementation settings used for CMU-MOSI and CMU-MOSEI.

Setting	CMU-MOSI	CMU-MOSEI
Serialized input	aligned_50.pkl, word-aligned	aligned_50.pkl, word-aligned
Text encoder / dimension	bert-base-uncased, 768; end-to-end fine-tuning	bert-base-uncased, 768; end-to-end fine-tuning
Acoustic / visual dimensions	25 / 171	25 / 171
Fixed sequence length	45	75
Loss / optimizer	MSE / AdamW	MSE / AdamW
Batch size	64	64
Learning rate / weight decay	10^–4^ / 10^–4^	10^–4^ / 10^–4^
Maximum epochs / early-stopping patience	200 / 40	200 / 40
Checkpoint criterion	Lowest validation MAE	Lowest validation MAE
Graph-attention depth	7 layers	4 layers
Repeated runs	5 random seeds	5 random seeds
Numeric handling	float32; acoustic −∞ replaced by 0; no additional feature normalization	float32; acoustic −∞ replaced by 0; no additional feature normalization
Training environment	Python 3.10.0; PyTorch 2.4.1 + cu118; CUDA 11.8; cuDNN 90100; RTX 4080 SUPER	Python 3.10.0; PyTorch 2.4.1 + cu118; CUDA 11.8; cuDNN 90100; RTX 4080 SUPER

### Evaluation protocol and metrics

We evaluate continuous and categorical sentiment prediction using mean absolute error (MAE), Pearson correlation (Corr), five-class accuracy (Acc-5), seven-class accuracy (Acc-7), and binary accuracy under Has0 and Non0 conventions. Has0 retains zero-label samples and separates negative from non-negative instances, whereas Non0 excludes zero-label samples and separates negative from positive instances. Acc-7 is calculated by clipping scores to [−3,3] and rounding to the nearest ordered class. Test data are used only for final evaluation; checkpoint selection uses validation MAE. All displayed metrics for a run are taken from the same selected checkpoint.

### Ethics statement

This study involved secondary analysis of publicly available benchmark datasets and did not involve recruitment of participants or collection of new human-subject data. No additional ethics approval or informed consent was required for this study. The person shown schematically in Fig 1 is an author-created illustration and does not depict a study participant or any identifiable individual.

## Results

### Comparative experimental results and analysis

We compare the proposed framework with TFN [6], LMF [8], MulT [9], MISA [10], CENet [11], LF-DNN as included in the CH-SIMS benchmark framework [28], and ALMT [21]. The supplied experiment package reports five-run means and sample standard deviations under the same metric implementation. Because paired sample-level predictions were not available for every baseline, numerical rank differences are treated as descriptive rather than as formal superiority tests. Tables 5 and 6 report the corresponding five-run results for CMU-MOSI and CMU-MOSEI, respectively.

**Table 5:** Five-run results on CMU-MOSI (mean ± sample SD).

Method	MAE↓	Corr↑	Has0 Acc-2↑	Non0 Acc-2↑	Acc-5↑	Acc-7↑
CENet	0.7654±0.0108	0.7353±0.0075	0.8053±0.0080	0.8121±0.0084	0.5087±0.0190	0.4490±0.0150
LF-DNN	0.9548±0.0190	0.6584±0.0120	0.7752±0.0105	0.7863±0.0108	0.3805±0.0180	0.3452±0.0155
LMF	0.9504±0.0195	0.6510±0.0125	0.7790±0.0102	0.7918±0.0105	0.3813±0.0178	0.3382±0.0150
MISA	0.7765±0.0135	0.7781±0.0082	0.8184±0.0085	0.8354±0.0087	0.4708±0.0195	0.4137±0.0165
MulT	0.8799±0.0165	0.7022±0.0100	0.7971±0.0093	0.8098±0.0095	0.4268±0.0190	0.3691±0.0162
TFN	0.9473±0.0180	0.6733±0.0115	0.7799±0.0098	0.7908±0.0101	0.3939±0.0185	0.3446±0.0158
ALMT	0.7960±0.0118	0.7140±0.0084	0.8200±0.0084	0.8100±0.0088	0.4877±0.0185	0.4058±0.0154
**Ours**	0.7290±0.0099	0.7880±0.0076	0.8233±0.0078	0.8373±0.0083	0.5230±0.0211	0.4561±0.0160

**Table 6:** Five-run results on CMU-MOSEI (mean ± sample SD).

Method	MAE↓	Corr↑	Has0 Acc-2↑	Non0 Acc-2↑	Acc-5↑	Acc-7↑
CENet	0.5959±0.0052	0.7175±0.0050	0.7852±0.0075	0.8138±0.0048	0.5215±0.0070	0.5126±0.0060
LF-DNN	0.5802±0.0070	0.7087±0.0065	0.8060±0.0085	0.8274±0.0065	0.5197±0.0080	0.5083±0.0070
LMF	0.5757±0.0072	0.7169±0.0064	0.8054±0.0082	0.8348±0.0062	0.5299±0.0078	0.5059±0.0068
MISA	0.5575±0.0060	0.7515±0.0057	0.8067±0.0080	0.8067±0.0055	0.5363±0.0075	0.5105±0.0065
MulT	0.5593±0.0063	0.7331±0.0060	0.8015±0.0083	0.8263±0.0058	0.5418±0.0074	0.5211±0.0064
TFN	0.5726±0.0065	0.7141±0.0062	0.7850±0.0095	0.8189±0.0068	0.5310±0.0078	0.5160±0.0068
ALMT	0.5790±0.0057	0.7360±0.0054	0.7870±0.0080	0.8160±0.0052	0.5421±0.0071	0.5005±0.0062
**Ours**	0.5541±0.0053	0.7551±0.0058	0.8139±0.0122	0.8473±0.0051	0.5450±0.0072	0.5269±0.0064

The quantitative comparisons show where the numerical gains are concentrated and where the margins remain small. On CMU-MOSI, the proposed model has a mean MAE of 0.7290, which is 0.0364 lower than the next-lowest value (CENet, 0.7654), and a Corr of 0.7880, which is 0.0099 higher than MISA (0.7781). Its Acc-7 is 0.4561 versus 0.4490 for CENet. The margins are narrower for binary accuracy: Has0 Acc-2 is 0.8233 versus 0.8200 for ALMT, and Non0 Acc-2 is 0.8373 versus 0.8354 for MISA. On CMU-MOSEI, the corresponding differences from the strongest baseline mean are also modest for several metrics: MAE is 0.5541 versus 0.5575 for MISA, Corr is 0.7551 versus 0.7515 for MISA, Acc-5 is 0.5450 versus 0.5421 for ALMT, and Acc-7 is 0.5269 versus 0.5211 for MulT. Thus, the proposed method is numerically favorable across the displayed mean metrics, but several margins are small. Because matched sample-level predictions are unavailable for every baseline, these differences are treated as descriptive comparisons rather than evidence that the method statistically outperforms existing approaches.

The earlier single-run comparison suggested a different MOSI–MOSEI pattern. Under the uniform five-run results supplied for this revision, that pattern did not persist. We therefore report the revised statistics and avoid a post hoc causal explanation based only on dataset scale or heterogeneity.

### Analysis of the training process

Fig 3 summarizes the five-seed training trajectories. The main decrease in training loss and validation MAE occurs early on both datasets. MOSI validation Corr and Acc-5 then approach a broad plateau, with occasional seed-specific spikes. MOSEI also converges early, but its validation Corr and Acc-5 show a mild later decline while training loss continues to decrease. This train–validation separation is consistent with later-stage overfitting, especially on MOSEI, and supports validation-based early stopping.

**Fig 3:**
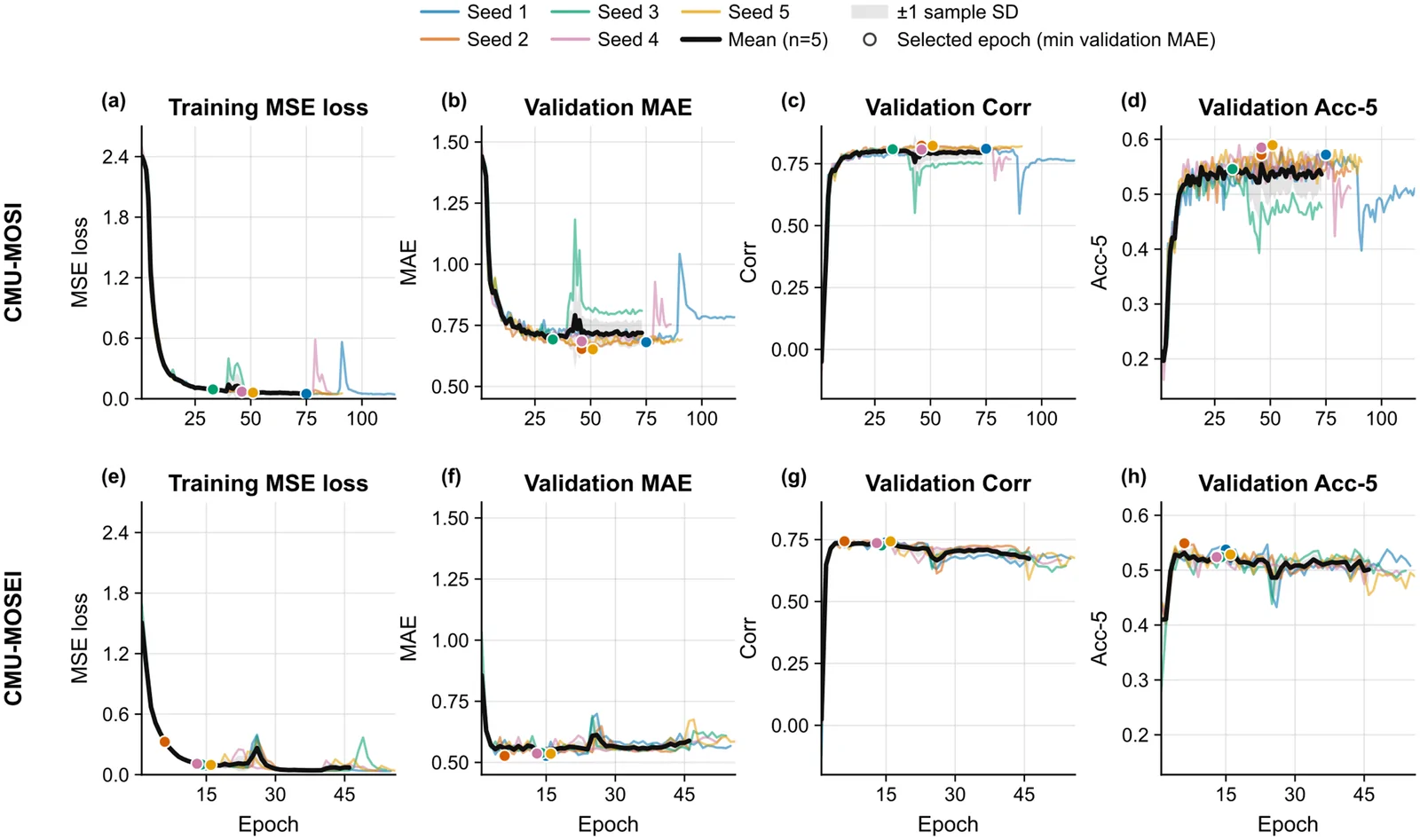
Five-seed training dynamics for CMU-MOSI (top row) and CMU-MOSEI (bottom row). Panels show training mean-squared error, validation MAE, validation correlation, and validation Acc-5. Thin colored lines represent seeds, the black line is their mean, the gray band is one sample standard deviation, and circles mark the validation-MAE-selected checkpoint for each seed.

### Ablation studies

To examine the contribution of individual components, we removed attention pooling, BiGRU encoding, and the static hyper-prior. Table 7 reports five-run means and sample standard deviations. Removing attention pooling produced the largest MOSI Corr decrease (0.7880 to 0.6735) and a substantial MOSEI Acc-7 decrease (0.5269 to 0.4130), consistent with its temporal-selection role. Removing BiGRU had a smaller but consistent effect on the regression-oriented metrics. Removing the static prior modestly affected MOSI MAE and Corr but more strongly affected MOSEI MAE and Acc-7. These patterns are consistent with complementary roles for temporal selection, sequential encoding, and prior conditioning; they are component-level associations rather than causal localization.

**Table 7:** Five-run component ablations on CMU-MOSI and CMU-MOSEI (mean ± sample SD).

Variant	CMU-MOSI				CMU-MOSEI			
	Has0 Acc-2↑	Acc-7↑	MAE↓	Corr↑	Has0 Acc-2↑	Acc-7↑	MAE↓	Corr↑
w/o Attention Pooling	0.7720±0.0095	0.4180±0.0185	0.7470±0.0125	0.6735±0.0094	0.8045±0.0130	0.4130±0.0075	0.6635±0.0063	0.6645±0.0070
w/o BiGRU	0.8055±0.0090	0.4310±0.0172	0.7400±0.0113	0.7365±0.0088	0.7785±0.0125	0.4075±0.0069	0.5995±0.0058	0.7098±0.0062
w/o Static Hyper-prior	0.8048±0.0085	0.4130±0.0168	0.7335±0.0107	0.7710±0.0084	0.7998±0.0124	0.4395±0.0068	0.6980±0.0056	0.7300±0.0061
**Full Model**	0.8233±0.0078	0.4561±0.0160	0.7290±0.0099	0.7880±0.0076	0.8139±0.0122	0.5269±0.0064	0.5541±0.0053	0.7551±0.0058

Complete Acc-2, Acc-5, Acc-7, MAE, and Corr vectors were retained for every run; a compact metric subset is shown here.

### Injection-position validation

Table 8 directly compares injection before GAT propagation, injection after the GAT stack but before readout, and no injection. On MOSI, pre-GAT injection had higher Corr and Acc-7 than both controls, while its MAE was nearly identical to post-GAT and lower than no injection. On MOSEI, pre-GAT had the lowest MAE, highest Corr, and strongest binary accuracies, while post-GAT had a slightly higher Acc-7 (0.5275 versus 0.5269). Thus, pre-propagation injection provided the strongest overall regression-oriented balance but was not uniformly best on every metric. The supplied pre-GAT and control runs used different seed identities, so this comparison is not treated as a paired significance test.

**Table 8:** Five-run injection-position comparison (mean ± sample SD).

Dataset	Position	Has0 Acc-2↑	Non0 Acc-2↑	Acc-5↑	Acc-7↑	MAE↓	Corr↑
MOSI	Pre-GAT	0.8233±0.0078	0.8373±0.0083	0.5230±0.0211	0.4561±0.0160	0.7290±0.0099	0.7880±0.0076
MOSI	Post-GAT	0.8004±0.0033	0.8187±0.0057	0.5069±0.0132	0.4108±0.0120	0.7299±0.0081	0.7487±0.0044
MOSI	No injection	0.7951±0.0065	0.8149±0.0068	0.5186±0.0041	0.4064±0.0038	0.7969±0.0077	0.7073±0.0067
MOSEI	Pre-GAT	0.8139±0.0122	0.8473±0.0051	0.5450±0.0072	0.5269±0.0064	0.5541±0.0053	0.7551±0.0058
MOSEI	Post-GAT	0.7937±0.0180	0.8071±0.0073	0.5330±0.0018	0.5275±0.0013	0.5750±0.0034	0.7383±0.0031
MOSEI	No injection	0.7476±0.0099	0.7694±0.0066	0.5033±0.0052	0.5173±0.0043	0.5864±0.0052	0.7189±0.0038

### Mechanism diagnostics and representative cases

The gate analysis pooled the complete test set across five pre-GAT seeds. Mean text/acoustic/visual gates were 0.369/0.262/0.216 on MOSI and 0.314/0.246/0.220 on MOSEI. This ordering indicates modality-selective rather than identical injection. In the first GAT layer, pooled attention mass was higher for text-related units (0.515 on MOSI and 0.489 on MOSEI) than for acoustic- or visual-related units. From the second layer onward, the pooled means approached one third. These values describe aggregate tendencies and do not imply uniform attention for every sample or head.

The dynamic hypernode also varied across samples. Its first two principal components explained 72.65% of variance on MOSI and 65.25% on MOSEI. For the final fused representation, the corresponding totals were 99.58% and 97.18%. These variance profiles establish that the hypernode is not a constant vector, but they do not by themselves establish sentiment-class separation or causal mediation. Fig 4 summarizes these results. A separate temporal-attention audit found that the current pooling implementation did not explicitly mask padded text positions; temporal-attention mass is therefore not used here as affirmative mechanism evidence.

**Fig 4:**
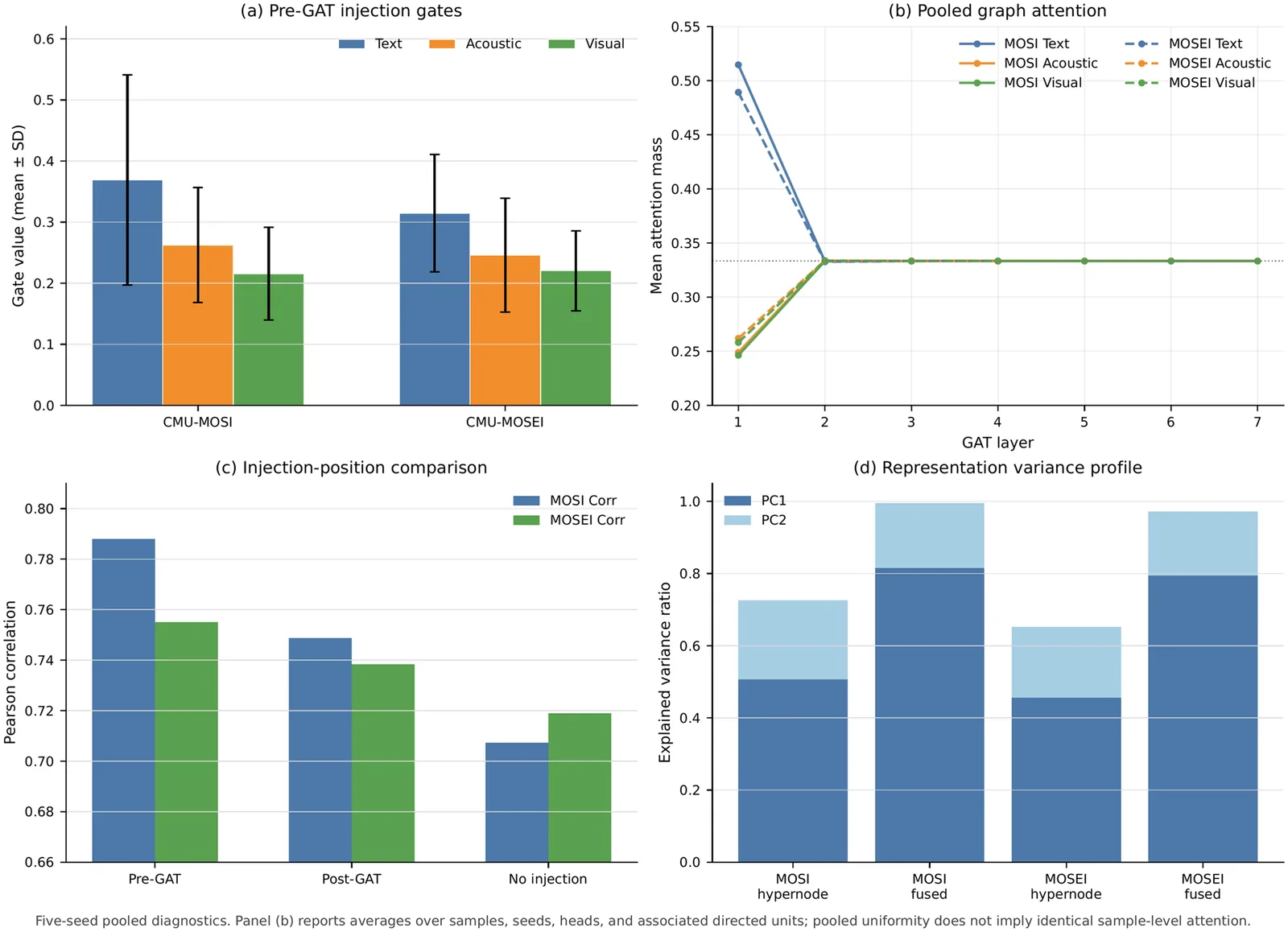
Five-seed mechanism diagnostics. (a) Mean modality-specific pre-GAT gate values; error bars show sample standard deviations across the pooled test-set values. (b) Mean graph-attention mass pooled over samples, seeds, heads, and associated directed units; the dotted line marks uniform mass (1/3), and the MOSEI trajectories stop at layer 4 because the final MOSEI model uses four GAT layers. (c) Mean Pearson correlation across five runs for pre-GAT, post-GAT, and no injection; the corresponding sample standard deviations are reported in Table 8. (d) Variance explained by the first two principal components of the dynamic hypernode and fused representation. Aggregate attention patterns and PCA variance do not imply identical sample-level behavior or causal mediation.

Balanced case selection included examples where pre-GAT helped, where it hurt, and where all variants failed. Table 9 reports six such cases from the common seed-7 diagnostic run. The mixed pattern shows that injection position changes individual predictions but is not beneficial for every segment.

**Table 9:** Balanced representative cases from the seed-7 diagnostic comparison.

Dataset	Case type	Sample ID	Label	Pre-GAT	Post-GAT	None
MOSI	Pre benefit	wMbj6ajWbic$_$5	0.2000	−0.4272	2.5891	2.7234
MOSI	Pre cost	zhpQhgha_KU$_$19	−0.8000	−1.8979	−0.7630	−1.0583
MOSI	Common error	nbWiPyCm4g0$_$2	−2.4000	0.2672	0.5326	−1.2266
MOSEI	Pre benefit	2W-U94hXuK0$_$4	−2.0000	−1.2036	−0.1739	−0.9763
MOSEI	Pre cost	102389$_$7	−1.0000	0.7560	−1.2943	−0.9817
MOSEI	Common error	111881$_$9	−2.0000	−0.5202	1.0053	−0.6302

### Effect of the number of graph fusion layers

The original development-stage depth sweep compared one to ten GAT layers. Because the model is trained for continuous sentiment regression and validation MAE is used for checkpoint selection, the final depth was chosen by prioritizing the joint MAE–Corr profile while also checking Acc-7 rather than by maximizing binary F1 alone. On CMU-MOSI, seven layers produced the lowest MAE (0.684), the highest Corr (0.813), and the highest Acc-7 (49.7%) among the tested depths, although five layers gave higher binary F1. On CMU-MOSEI, four layers produced the lowest MAE (0.539), the highest Corr (0.768), and the highest Acc-7 (53.8%), while other depths produced higher binary F1. These observations motivated the seven-layer MOSI and four-layer MOSEI configurations used in the five-seed main and injection-position experiments. The depth sweep itself was a development-stage sensitivity analysis without repeated-seed evaluation, so these choices should be interpreted as dataset-specific settings for the present experiments, not as evidence of a universally optimal GAT depth. Table 10 summarizes the development-stage graph-depth results for both datasets.

**Table 10:** Impact of different graph fusion depths on the CMU-MOSI and CMU-MOSEI datasets.

Layers	CMU-MOSI				CMU-MOSEI			
	F1 (H/N)↑	Acc-7↑	MAE↓	Corr↑	F1 (H/N)↑	Acc-7↑	MAE↓	Corr↑
1	82.9/84.5	45.4	0.712	0.796	81.7/85.4	53.3	0.550	0.757
2	81.7/83.4	48.5	0.697	0.802	80.4/84.7	53.5	0.545	0.759
3	82.6/84.5	45.7	0.702	0.800	82.0/84.9	53.3	0.541	0.759
**4**	82.4/84.3	49.0	0.694	0.801	**79.8/84.7**	**53.8**	**0.539**	**0.768**
5	83.6/85.8	47.4	0.695	0.804	81.5/85.3	52.8	0.548	0.761
6	83.4/85.3	47.6	0.703	0.796	82.1/84.9	52.6	0.547	0.751
**7**	**83.0/84.9**	**49.7**	**0.684**	**0.813**	82.6/84.5	52.6	0.546	0.766
8	83.1/84.8	45.9	0.701	0.798	82.3/85.2	52.4	0.549	0.760
9	82.3/84.3	46.5	0.717	0.787	81.7/85.6	52.8	0.550	0.758
10	83.0/84.6	47.8	0.689	0.801	81.6/85.0	53.5	0.547	0.755

F1 (H/N) reports Has0/Non0 F1. All entries in each row are taken from the same evaluation epoch; bold text indicates the depth adopted for that dataset.

## Discussion

The experimental results show that the position of hypernode injection has a measurable effect on multimodal sentiment prediction. Across the five validation-selected runs, injecting the hypernode before graph propagation produced the most consistent overall performance on both CMU-MOSI and CMU-MOSEI, particularly for regression-oriented metrics. Post-GAT injection achieved a slightly higher Acc-7 score on MOSEI, indicating that the advantage of pre-GAT injection is not uniform across all evaluation criteria. A more appropriate interpretation is therefore that early global conditioning provides a favorable balance under the present experimental setting. By introducing the shared multimodal context before message passing, the graph layers can propagate representations that have already been adjusted according to sample-level information, rather than incorporating this information only after graph interaction has taken place.

The diagnostic analyses provide further insight into how the model uses this global conditioning. Text consistently received the largest average injection gate on both datasets, suggesting that the model relies more strongly on textual information when adjusting modality-specific representations. At the same time, the dynamic component of the hypernode varied across samples, which indicates that the injected context is not reduced to a fixed global bias. The first GAT layer also assigned relatively greater attention to text-related units, whereas attention distributions became more balanced in deeper layers. Taken together, these patterns suggest that the model first emphasizes the modality carrying the strongest sentiment signal and then progressively integrates information across modalities during graph propagation. This interpretation is consistent with the known importance of language in multimodal sentiment analysis, while still allowing visual and acoustic cues to modify the final representation.

These diagnostic results should nevertheless be interpreted with some caution. Average gate values and graph-attention weights describe how information is distributed inside the trained model, but they do not by themselves demonstrate causal mediation between particular modalities and the final prediction. Similarly, variation observed in the dynamic hypernode through PCA reflects changes in its representation across samples rather than explicit class separation. The temporal-attention analysis revealed an additional limitation: a noticeable proportion of attention was assigned to padded positions because padding was not explicitly masked during temporal pooling. For this reason, temporal-attention maps are not treated as evidence for the interpretation of model behavior in the present study. A padding-aware pooling mechanism would provide a cleaner basis for such analysis in future implementations.

The current results also define the practical scope of the proposed framework. Accordingly, the conclusions should be interpreted as specific to CMU-MOSI and CMU-MOSEI, the complete word-aligned tri-modal input setting, and the reported training and evaluation protocol. The model is designed for settings in which text, visual, and acoustic streams are simultaneously available and can be aligned reliably at the word or segment level. Under these conditions, the compact graph provides an efficient way to combine modality-specific information with a sample-dependent global context. The present experiments, however, do not examine incomplete modalities, severe corruption, additive noise, multilingual inputs, unaligned streams, cross-domain transfer, or multi-turn conversational structure. These scenarios introduce different sources of uncertainty and may require more explicit mechanisms for modality reliability or temporal context. The current hypernode also uses simple mean-based aggregation of the three pooled modality representations. Although this choice keeps the architecture compact, attention-weighted or uncertainty-aware aggregation may provide a more flexible alternative when modality quality varies substantially across samples.

Several experimental limitations should also be considered when interpreting the reported gains. The available experimental archive did not preserve the exact CMU-MultimodalSDK release or the original versions of the acoustic and visual feature extractors, which restricts complete reconstruction of the preprocessing environment. In addition, the pre-GAT and control configurations were each evaluated over five runs, but they were not generated from an identical set of random seeds. Their comparison therefore reflects repeated-run performance rather than a strictly paired significance test. The graph-depth analysis was also conducted without repeated-seed evaluation, so the observed degradation at greater depth should not be taken as direct evidence of graph oversmoothing. Finally, computational efficiency was not evaluated systematically, and the current experiments do not determine whether similar performance could be obtained with fewer graph layers, reduced hidden dimensions, or sparse attention mechanisms.

Future work should address these limitations through a more tightly controlled evaluation protocol. Positional ablations should be repeated with identical preregistered seeds, and paired statistical tests should be reported when predictions from matched runs are available. Padding-aware temporal pooling is also necessary before temporal attention can be used reliably for interpretation. Beyond these implementation issues, robustness to modality dropout, corrupted inputs, and domain shift deserves particular attention because real multimodal systems rarely receive equally reliable signals from every modality. Comparing mean-based hypernode construction with attention-weighted and uncertainty-aware alternatives may clarify whether the global representation can adapt more effectively to such variation. Lightweight or sparse graph-attention variants could further reduce computational cost. Extensions to multilingual sentiment analysis, multi-turn conversation, and cross-domain transfer would also help establish whether the same global-conditioning mechanism remains useful beyond aligned English-language segment-level benchmarks. In this context, incomplete-modality approaches such as proxy-driven robust multimodal fusion provide a relevant direction for comparison [29].

## Conclusions

We proposed a multimodal sentiment framework that combines BiGRU temporal modeling, attention pooling, dynamic hypernode injection, and graph-attention fusion. The sample-specific hypernode and static prior are injected through modality-specific gates before graph propagation.

Across five validation-selected runs, the model achieved an MAE of 0.7290±0.0099, Corr of 0.7880±0.0076, and Acc-7 of 0.4561±0.0160 on MOSI. On MOSEI, the corresponding values were 0.5541±0.0053, 0.7551±0.0058, and 0.5269±0.0064. The pre/post/no-injection comparison showed the strongest overall regression-oriented balance for pre-GAT injection, although it did not lead on every metric. Gate, graph-attention, PCA, and balanced case analyses further showed that the injected prior was sample dependent and modality selective.

These findings support pre-propagation hypernode injection within complete, aligned tri-modal segment-level data. They do not establish universal superiority, robustness to missing or corrupted modalities, or generalization to multilingual, cross-domain, or multi-turn settings.

## Supporting information

S1 Data Numerical values reported in the results tables. (XLSX)
